# Multi-omics disease module detection with an explainable Greedy Decision Forest

**DOI:** 10.1038/s41598-022-21417-8

**Published:** 2022-10-07

**Authors:** Bastian Pfeifer, Hubert Baniecki, Anna Saranti, Przemyslaw Biecek, Andreas Holzinger

**Affiliations:** 1 Institute for Medical Informatics Statistics and Documentation, Medical University Graz, Graz, Austria; 2 MI2DataLab, Faculty of Mathematics and Information Science, Warsaw University of Technology, Warsaw, Poland; 3 Human-Centered AI Lab, University of Natural Resources and Life Sciences, Vienna, Austria; 4 Alberta Machine Intelligence Institute, Alberta, Canada

**Keywords:** Predictive markers, Data integration, Machine learning

## Abstract

Machine learning methods can detect complex relationships between variables, but usually do not exploit domain knowledge. This is a limitation because in many scientific disciplines, such as systems biology, domain knowledge is available in the form of graphs or networks, and its use can improve model performance. We need network-based algorithms that are versatile and applicable in many research areas. In this work, we demonstrate subnetwork detection based on multi-modal node features using a novel Greedy Decision Forest (GDF) with inherent interpretability. The latter will be a crucial factor to retain experts and gain their trust in such algorithms. To demonstrate a concrete application example, we focus on bioinformatics, systems biology and particularly biomedicine, but the presented methodology is applicable in many other domains as well. Systems biology is a good example of a field in which statistical data-driven machine learning enables the analysis of large amounts of multi-modal biomedical data. This is important to reach the future goal of precision medicine, where the complexity of patients is modeled on a system level to best tailor medical decisions, health practices and therapies to the individual patient. Our proposed explainable approach can help to uncover disease-causing network modules from multi-omics data to better understand complex diseases such as cancer.

## Introduction

Network-based algorithms are utilized in most areas of research and industry. They find their application in virtually all areas, from agriculture to zoology, and are particularly useful in systems biology^1,2^. Networks are very important to solve problems in decision making and knowledge discovery^3^. Such networks can represent many real-world phenomena and are technically described by graphs, which provide a unifying abstraction by which such real-world networks can be represented, explored, predicted and discovered^4^. Most importantly, such structures help to make complex phenomena re-traceable, transparent, interpretable and thus explainable to human experts. Better interpretability promotes developer understanding. However, it also means easier understanding and better explanation of future predictions for end users, thus promoting reasonable and data-driven decisions to make personalized choices that can ultimately lead to higher quality healthcare services^5^.

What we need in the future are context-adaptive methods, i.e. systems that construct contextual explanatory models for classes of real-world phenomena. This is a goal of so-called explainable AI^6^, which is actually not a new field; rather, the problem of explainability is as old as AI itself. While the rule-based approaches of early AI were comprehensible “glass box” approaches, at least in narrow domains, their weakness lay in dealing with the uncertainties of the real world^7^. Actionable Explainable AI (AXAI) is intended to help promote trust-building features by bringing decision analytic perspectives and human domain knowledge directly into the AI pipeline^8,9^.

In this work, we focus as example application on systems biology with a particular emphasis on biomedicine. Recent developments in bioinformatics and machine learning have made it possible to analyze huge amounts of biomedical data, paving the way for future precision medicine^10^. A grand goal of precision medicine is in modeling the complexity of patients to tailor medical decisions, health practices and therapies to the individual patient. To reach this goal efficient computational methods, algorithms and tools are needed to discover knowledge and to interactively gain insight into the data^11^. Network-based approaches help uncover disease-causing interactions between genes to better understand diseases such as cancer^12–14^. In systems biology, for instance, network analyses are performed for protein–protein co-expression networks as well as for metabolomics data through metabolomic pathway analyses.

Recent evidence suggests that complex diseases, such as cancer, need to be studied in a multi-modal feature space comprising multiple biological entities (multi-omics), as diverse components contribute to a single outcome^15,16^. Multi-omics clustering methods are successfully utilized to detecting disease subtypes, that is patient groups with similar molecular characteristics. Recently developed multi-omics clustering approaches include SNF^17^, PINSplus^18^, and HC-fused^19^.

Another typical research goal is the discovery of disease-causing genes to efficiently monitor disease progression^10^. These so-called biomarkers are often detected from multi-modal omics data sets and here it is important to allow physicians to interactively intervene, read out corresponding patterns from the data and adjust their treatment accordingly.

Feature selection (FS) algorithms are often used to detect such patterns. One popular family of FS algorithms is based on the random forest (RF) classifier. RFs can efficiently handle heterogeneous data types, don’t need normalization of their values^20^, compute feature importances and are therefore particularly suited for the multi-modal case. One example of a popular RF-based feature selector is the Boruta algorithm^21^. The heuristic introduced by the authors quantifies the importance of a feature by the loss of accuracy of classification caused by randomly permuted probes (*shadow features*) of the original feature set. Recent work combines the Boruta algorithm with Shapley values^22^. Another RF-based feature selection algorithm is introduced in^23^. The authors have developed a regularized random forest approach that penalizes the selection of a new feature for splitting a node when its information gain is similar to the features used in previous splits.

However, most of these methods treat genes/features independently or do not account for any dependencies between variables. Contrarily, approaches which do account for dependencies between genes are tailored towards the detection of network modules with correlated features only (like in co-expression analyses). They typically utilize community detection algorithms^24^ or unsupervised clustering. Disease-causing genes, however, may function in a multivariate manner without necessarily being correlated.

Most recently, the machine learning community has developed deep learning techniques which can be applied to networks comprising edge and node features of arbitrary dimensions. Deep neural networks like DeepOmix^14^ incorporate data from various sources to predict time-to-event in survival analysis. Graph neural networks (GNNs)^25,26^ are very versatile^27^ and will certainly have a big impact on systems biology research. A major difficulty with these approaches is that they are considered black-box models. The decisions they make cannot be traced back precisely, therefore medical doctors may not trust decisions made by black-box models. To address this shortcoming, first explainable GNN methods are available and are currently under strong development. Examples include GNNexplainer^28^, PGExplainer^29^, and GNN-LRP^30^. GNNExplainer provides *local* explanations for predictions of any graph-based model. It can be applied to node classification as well as graph classification tasks. PGExplainer is a parameterized modification of the GNNexplainer. In contrast to the GNNexplainer, it provides explanations on a model level; especially useful for graph classification tasks. The GNN-LRP approach is derived from higher-order Taylor expansions based on Layer-wise relevance (LRP). It explains the prediction by extracting paths from the input to the output of the GNN model that contributes the most to the prediction. These paths correspond to *walks* on the input graph. GNN-LRP was developed for explanations on the node level but was recently modified to also work for graph classification in a special application set-up^31^.

The aforementioned methods may facilitate the discovery of disease-causing regions within biological networks, but not directly can be used for network module *selection* purposes. Explainers on GNNs usually highlight the importance of edges, nodes or walks within the network, but they do not rank whole subnetworks by their importance. Also, methods for explanations are often black-box models themselves, and thus may not be trustworthy either. In addition, standard GNN architectures cannot handle multi-graphs which may hinder the analysis for incomplete data, which is often the case in the biomedical domain.

At the same time, only a few FS methods are applicable on network structured input data. Some developments in this direction are realized within the R-packages glmgraph^32^ The implemented method uses a regularization term to incorporate network topology into the feature selection process. The underlying algorithm, however, does not function on the network itself.

In this work, we present a Greedy Decision Forest (GDF) for the selection of disease network modules. The proposed algorithm directly functions on a network by sampling node features by random walks. A set of decision trees are derived from the visiting nodes, ultimately forming the decision forest. A greedy process on the selection process of the decision trees is initiated, and the algorithm converges to a set of subnetworks. In the following we will use the term “network module” and “subnetwork” interchangeable.

Our proposed methodology naturally handles multi-modal data with a high degree of interpretability, thereby perfectly suited for multi-omics types of analyses in the biomedical domain.

## Greedy Decision Forest

**Figure 1:**
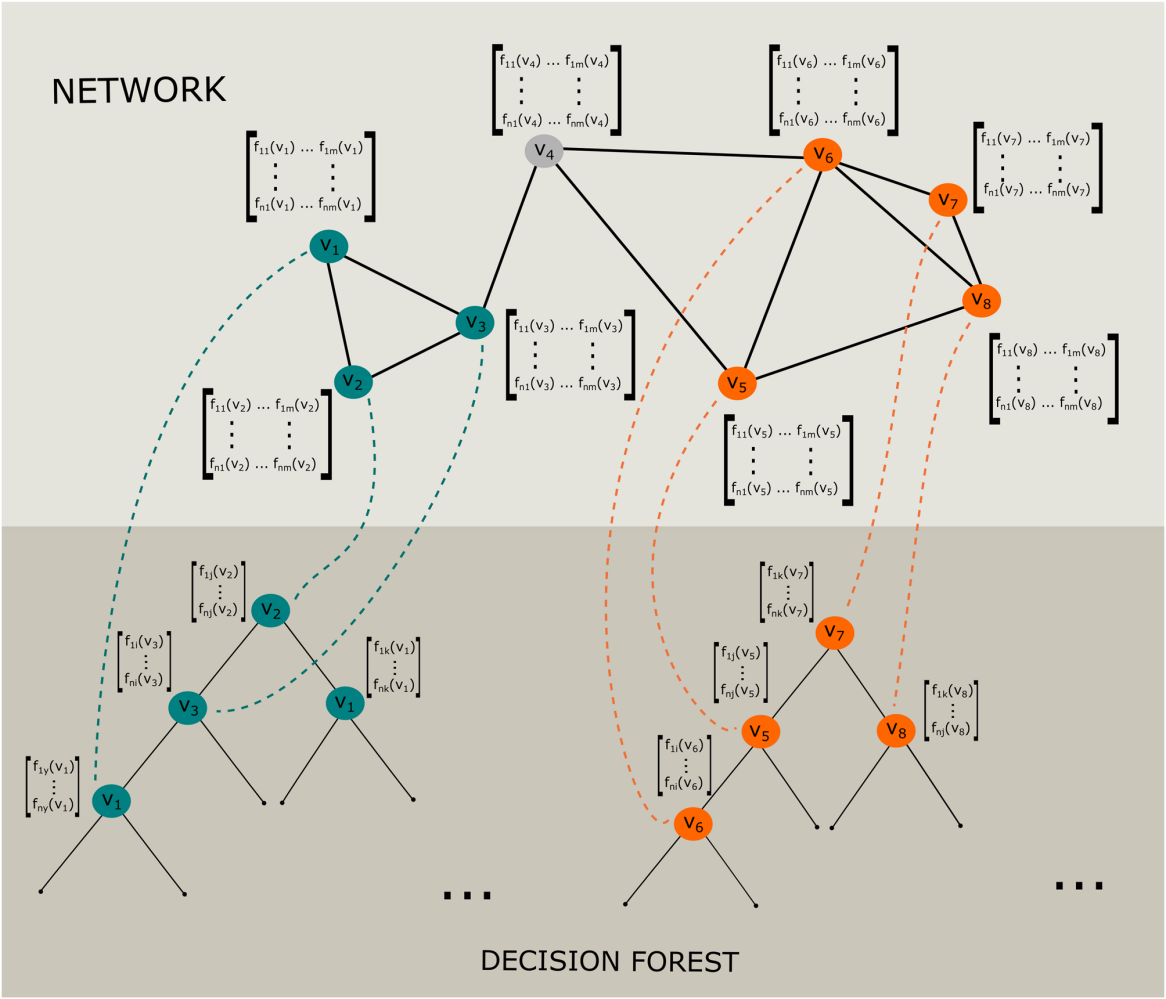
Deriving a Decision Forest from a network comprising multi-modal node feature vectors. Displayed is the network at the top and the derived decision forest at the bottom. Each node of the network comprises *m* node feature vectors of length *n*. Each random walk creates a *mtry* set of feature vectors used for building a decision tree.

The core functionality of our proposed network module detection approach includes a tree-building process derived from a network $G=(V,E)$. Each node $\mathbf {v}\in V$ comprises node feature vectors $f(\mathbf {v})$ of arbitrary dimensions (see Fig. 1). In order to build a single tree we randomly select a node $\mathbf {v} \in V$ located on the network *G*. From that node a random walk is initialized. The depth of this random walk is set to $\sqrt{|V|}$, which is the squared root of the total number nodes. This value is frequently used as a rule-of-thumb, and a default value in popular R-packages like ranger^33^ and randomForest^34^. It is the so-called *mtry* parameter, and it specifies the number of features a decision tree includes. In our case, features of visited nodes by the random walk are in the *mtry* set. The described procedure is repeated until *ntree* decision trees are generated. Figure 1 illustrates this process. A network is shown with eight nodes. Each of these nodes has *m* node feature vectors of length *n*, where *n* is the number of samples and *m* is the number of modalities. Two random walks of depth four are highlighted. Each of these walks forms a Decision Tree (blue and orange nodes). It should be noted, that the random walk may visit the exact same node multiple times. So is the case for the first random walk. The derived Decision Tree contains the node $v_{1}$ twice. Thus, the associated features are used twice for splitting a tree node (Fig. 1: blue Decision Tree). Node $v_{4}$ was not captured by the random walks.

Based on the network-derived decision trees we start the proposed greedy algorithm for network module selection. At each greedy step, *ntree* decision trees are generated, while the selection of network nodes and their corresponding features depend on the outcome of previous iterations. High performing trees remain in the *ntree* set while low performing trees and their corresponding nodes and node features are eliminated.

In the following, we will introduce necessary notations and describe the greedy algorithm in great detail.

### Network module selection using a Greedy Decision Forest

We define a Decision Tree classifier as $T(x;\Theta, X)$, where $\Theta$ consists of a set of split rules and *X* includes the variables/features used for splitting. Given an input vector *x*, $T(x;\Theta, X)$ assigns a given data point to a specific class. An ensemble of Decision Tree classifiers is called Decision Forest (DF). It is defined as $\{T_{k}(x,\Theta _{k}, X_{k}), k=1,\ldots, ntree\}$, where $X_{k}$ is a set of randomly selected features from the input feature space the *k*-th Decision Tree $T_{k}$ is based on.

Let us further assume a graph $G=(V,E)$ is specified. The nodes $v\in V$ represent the features in *X* and the edges $(v_{i},v_{j}) \in E$ are reflecting any kind of dependency of these features. This graph for example could be a knowledge graph, which connects node features with domain-specific and domain-relevant relationships. An example of such graph is a Protein-Protein network, where lab-validated functionally interacting genes are connected.

Here, we propose to restrict the features $X_{k}$ of a single tree $T_{k}$ within the forest to be neighboring nodes within graph *G*. This regularization ensures that related features are located on the same Decision Tree. As a consequence, classifications made by this tree model might be more reliable and interpretable for the domain expert. Accordingly, our proposed Decision Forest is defined as $\{T_{k}(x,\Theta _{k}, X^{G}_{k}), k=1,\ldots, ntree\}$, where $X^{G}_{k}$ is a set of features determined by a random walk on graph *G*. Starting from that Decision Forest we execute the proposed greedy steps for tree-based module selection (see Algorithm 1). 
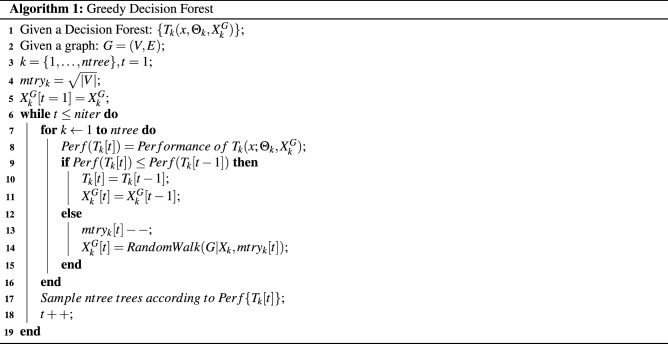


For each greedy step *t* we calculate the performance for all $k\in \{1,\ldots,ntree\}$ Decision Trees based on the *out-of-bag* samples (Algorithm 1: line 8). Here, we use ROC-AUC as a performance estimate, but this could be replaced by any type of performance measure such as accuracy (in the case of a balanced dataset) or mutual information^35^. In case the *out-of-bag* performance of the *k*-th Decision Tree $Perf(T_{k}[t])$ at greedy step *t* is lower than the performance of the Decision Tree from the previous iteration ($t-1$), the suggested Decision Tree and the corresponding node features are dropped. In case the Decision Tree provides better performance, a random walk on a subgraph, specified by the features in $X_{k}[t]$ at greedy step *t*, is initialized (Algorithm 1: line 14). Note, the depth of this walk is now decreased by one (Algorithm 1: line 13). Consequently, the algorithm aims for a minimal set of tree features while not decreasing its performance. After updating the Decision Trees, we sample a new set of *ntree* Decision Trees according to their *out-of-bag* performance values which initiates the mentioned selective process (Algorithm 1: line 17). The algorithm repeats the aforementioned procedure and terminates after *niter* greedy steps. The selected modules are represented by the node features $X^{G}_{k}$ at iteration $t=niter$.

The feature space *X* can naturally be extended to a multi-modal representation. It may simply consist of a set of matrices instead of a single matrix, representing the samples as rows and the features as columns. Figure 1 illustrates this scenario. Each node is represented by a feature matrix. Each column of this matrix is a feature vector from a specific modality, all associated with the same node. The tree building process is free to pick features from any of these given modalities for splitting a tree node, as long it is associated with the same network node.

Decision Forests are particularly suited for the multi-modal case, because DFs are scale invariant. There is no need for data normalization prior to execution and thus DFs provide an ideal framework for the analysis of heterogeneous input data. At the same time the node-split rules of a DT within the forest can be read out and therefore it is straightforward to re-trace the contribution of each modality to the final outcome.

### Network module importance scores

We compute an importance score for each of the selected modules. The proposed estimate is based on the network module *out-of-bag* performance $Perf(T_{k})$ at the *niter* greedy step, which is the performance of the Decision Tree associated with that specific module. However, $Perf(T_{k})$ estimate of a module does not depend on the number of features used by the derived Decision Tree. Thereby, redundant features do not affect the performance in a negative way. Here, we aim to infer the smallest possible module with maximal performance. Thus, an additional importance measure is needed to account for the importance of the actual edges forming the module. Edge importance is calculated as

1 $$\begin{aligned} IMP_{e}((\mathbf {v}_{i},\mathbf {v}_{j}) \in E)):= \sum _{t}^{niter}\sum _{k}^{ntree} Perf(T_{k}(\{\mathbf {v}_{i},\mathbf {v}_{j}\}\in X_{k}^G)[t]). \end{aligned}$$

The above equation assigns the performance of a module $X_{k}^G$ to the edges $(\mathbf {v}_{i},\mathbf {v}_{j})$ forming it. This is accomplished for all trees generated during the greedy steps *t*. Consequently, we not only account for the performances but also for the number of times an edge is part of the *ntree* set. As a result, less important edges are purified during the selective greedy process.

The normalized edge importance of a module is calculated as

2 $$\begin{aligned} \overline{IMP_{e}}(X_{m}^G):= \frac{\sum _{i,j} IMP_{e}((\mathbf {v}_{i},\mathbf {v}_{j})\in X_{m}^G)}{\# (\mathbf {v}_{i},\mathbf {v}_{j})\in X_{m}^G}, \end{aligned}$$

where $m\in \{1,\ldots,ntree\}$ and $(\mathbf {v}_{i},\mathbf {v}_{j}) \in E$, where $i,j \in \{1, \ldots, |V|\}$. The nominator reflects the cumulative edge importance score within the *m*-*th* module $X_{m}^G$, and is normalized by the number of edges forming that module.

Finally, module importance is calculated as

3 $$\begin{aligned} IMP_{m}(X_{m}^G):= \overline{IMP_{e}}(X_{m}^G) + Perf(T_{m}), \end{aligned}$$

where $Perf(T_{m})$ is the *out-of-bag* performance of the *m*-th DT associated with the module $X_{m}^G$. We utilize the $IMP_{m}$ to rank the obtained modules $X_{m}^G$ after *niter* greedy steps.

### Node feature importance scores

Our proposed Greedy Decision Forest allows to retrace from which modality the features were sampled to form the selected modules. The importance of these features can be calculated by standard tree-based importance measures, such as as the Gini impurity index^36^. The Gini index at tree node $\mathbf {v}^{t}$ is

4 $$\begin{aligned} Gini(\mathbf {v}^{t})=\sum _{c=1}^{C}\hat{p}_{c}^{\mathbf {v}^{t}}(1-\hat{p}_{c}^{\mathbf {v}^{t}}), \end{aligned}$$

where $\hat{p}_{c}^{\mathbf {v}^{t}}$ is the proportion of samples belonging to class *c* at tree node $\mathbf {v}^{t}$. The Gini information gain obtained by a feature $X_{i}$ for splitting node $\mathbf {v}^{t}$ is the difference between the Gini impurity at node $\mathbf {v}^{t}$ and the weighted average of impurities at each child node of $\mathbf {v}^{t}$. The Gini information gain is defined as

5 $$\begin{aligned} Gain(X_{i},\mathbf {v}^{t})=Gini(\mathbf {v}^{t})-w_{L}Gini(\mathbf {v}^{t}_{L},X_{i})-w_{R}Gini(\mathbf {v}^{t}_{R},X_{i}), \end{aligned}$$

where $\mathbf {v}^{t}_{L}$ and $\mathbf {v}^{t}_{R}$ are the left and right child nodes of $\mathbf {v}^{t}$, respectively, and $w_{L}$ and $w_{R}$ are the proportions of *c*-class instances assigned to the left and right child nodes. At each node $\mathbf {v}^{t}$, a set of features (the *mtry* set), and the feature with the maximum $Gain(X_{i}, \mathbf {v}^{t})$ is used for splitting the node. We calculate the importance of a feature within a detected module $X_{m}^G$ as

6 $$\begin{aligned} IMP_{f}(f(\mathbf {v}_{i}\in V)) =\sum _{t}^{niter}\sum _{k}^{ntree} Gain(f(\mathbf {v}_{i})\in X_{k}^G)[t], \end{aligned}$$

and it is normalized as

7 $$\begin{aligned} \overline{IMP_{f}}(f(\mathbf {v}_{i})\in X_{m}^G) = \frac{IMP_{f}(f(\mathbf {v}_{i})\in X_{m}^G)}{niter\cdot ntree}, \end{aligned}$$

where $f(\mathbf {v}_{i})$ refers to a feature vector associated with a network node $\mathbf {v}_{i}$. Similar to the calculation of the edge importance $IMP_{e}$, we measure the importance of a feature by accounting for the number of times it is a member of the *ntree* set of modules during the greedy process.

### The Greedy Decision Forest as an explainable classifier

We also consider our proposed GDF to be a machine learning model calculating predictions based on multi-modal data. We can explain the local behaviour of this model using SHapley Additive exPlanations (SHAP)^37^ derived from the Shapley value framework^38,39^, which is a state-of-the-art approach for explaining any predictive classifier^40^. A tree-based structure of GDF allows obtaining informative local and global explanations using the efficient TreeSHAP algorithm^41^.

Although SHAP values can be estimated for other machine learning models, e.g. neural networks, it is computationally beneficial to apply TreeSHAP in multi-omics studies, where classification based on a large number of features is typical. Furthermore, GDF aims to reduce the number of considered features at each iteration step, which makes the models’ interpretations faster to compute and easier to comprehend.

We aim to introduce a corresponding node feature importance score based on SHAP. This alternative further extends our understanding of GDF and promotes it as an explainable approach for biomedical knowledge discovery and decision making.

In general, SHAP value $sv_j(x)$ for a given greedy decision forest *g*, an observation *x* and variable *j* can be defined as^37,41^

8 $$\begin{aligned} sv_j(g,\;x) = \sum _{S \subseteq J\setminus \{j\}} \frac{|S|!(|J|-|S|-1)!}{|J|!} \left( \mathbf {E} [g(x)\;|\;x_{S \cup \{j\}}] - \mathbf {E} [g(x)\;|\;x_S]\right), \end{aligned}$$

where *S* is a subset of all variables *J*, |*S*| denotes its size, and $x_S$ represents the input values of variables in *S*. The latter are used to compute conditional expectation function of the model’s predictions for all possible variable orderings. SHAP values are additive, i.e.

9 $$\begin{aligned} g(x) = sv_0(g) + \sum _{j \in J} sv_j(g,\;x), \end{aligned}$$

where $sv_0$ is the baseline probability, conventionally an average prediction over a subset of data, and $sv_j(x)$ equals the *j*-th variable’s attribution to the prediction *g*(*x*). Consecutively, SHAP importance for variable *j* can be defined as an average of the absolute SHAP values per variables across the *N* observations in data

10 $$\begin{aligned} SVIMP_j(g) = \frac{1}{N}\sum _{x \in X^G_m} |sv_j(g,\;x)|. \end{aligned}$$

We note that this is a widely-used heuristic approach, while other aggregations might also be used to quantify feature importance. The main property of SHAP is their additivity with respect to features, which we utilize to aggregate the attributions across multi-modal data. Using the SHAP framework, node feature importance can be calculated as

11 $$\begin{aligned} SVIMP_f(f(\mathbf {v}_{i}) \in X_{m}^G) = \sum _{j \in f(\mathbf {v}_{i})} SVIMP_j, \end{aligned}$$

similarly to $IMP_f$. In fact, *g* can be a GDF, as well as a single decision tree, e.g. the best module classifier. Moreover, one can further extend $SVIMP_j$ and $SVIMP_f$ to represent the importance of feature pairs using SHAP interaction values derived from the Shapley interaction index^41^. We specifically incorporate the network structure into the greedy decision forest creation process to account for any potential feature relationships derived from domain knowledge.

Note that although a limitation of SHAP values is its inability to capture additive effects of correlated features^37^, the possible *interactions* encoded in a network are not necessarily *correlations* between features. Other feature attribution methods can be used in our framework, e.g. SHAPR^42^, which aims to provide more accurate approximations to Shapley values in situations when features are dependent.

## Results

### Synthetic data sets

We have exercised our approach on synthetic Barabasi networks^43^. Barabasi networks were developed to reflect real-world biological networks, where subsets of nodes (e.g genes) are strongly connected and organized as communities. They are scale-free networks and are generated using a preferential attachment mechanism. For each simulated network we randomly selected four connected nodes $\mathbf {v}_{1}, \mathbf {v}_{2}, \mathbf {v}_{3},$ and $\mathbf {v}_{4}$, which combined with their associated edges form a functional module. Each node is linked to a feature vector comprising binary feature values of 1000 samples. The feature values of all nodes are uniformly distributed, while the selected nodes have the following functional relationship

12 $$\begin{aligned} Module(V,E):= [f(\mathbf {v}_{1}) \wedge f(\mathbf {v}_{2})] \oplus [f(\mathbf {v}_{3}) \wedge f(\mathbf {v}_{4})], \end{aligned}$$

where $f(\mathbf {v})$ is the feature vector associated with node $\mathbf {v}$.

**Figure 2:**
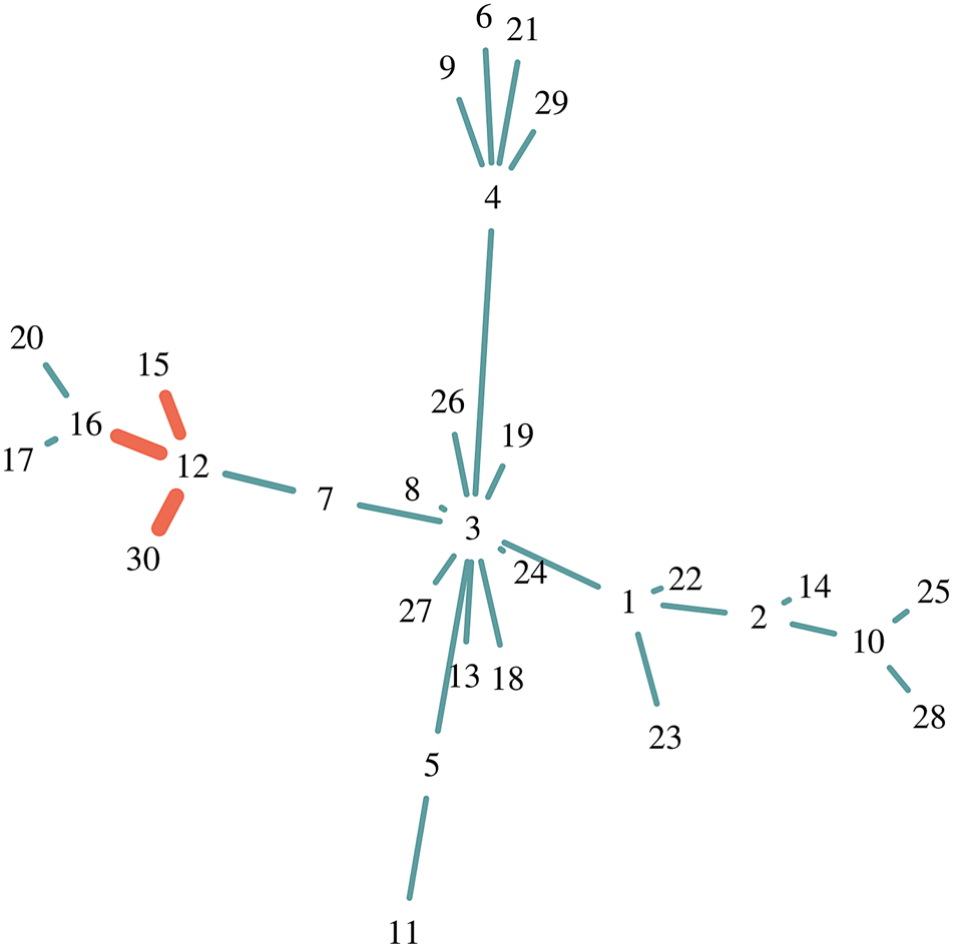
Simulated Barabasi network. The correctly identfied subnetwork ({$\mathbf {v}_{12}, \mathbf {v}_{15}, \mathbf {v}_{16},$, $\mathbf {v}_{30}$ }) is highlighted in red. The thickness of the edges is reflected by the $IMP_{e}$ edge importance values. The *out-of-bag* performance of the detected module is $Perf(T_{m})=1$, and the normalized edge importance score is $\overline{IMP_{e}}=0.67$. The overall importance score thus is $IMP_{m}=1.67$.

We have generated a Barabasi network comprising $|V|=30$ nodes with a power of the preferential attachment of 1.2 using the R-package igraph^44^. The selected nodes of the functional module are $\mathbf {v}_{12}, \mathbf {v}_{15}, \mathbf {v}_{16},$ and $\mathbf {v}_{30}$ and form a sub-graph. Figure 2 shows the resulting Barabasi Network and the top-1 module correctly verified by our approach (highlighted in red). The thickness of the edges reflects the importance of the edges determined by $IMP_{e}$.

Furthermore, Table 1 lists the selected modules sorted by their module importance scores ($IMP_{m}$). Overall, six unique modules were detected. As can be seen from Table 1, the first three modules have the same *out-of-bag* module performance ($Perf(T_{m})=1$). Their associated DTs perform equally well with an accuracy of $AUC=1$. However, these modules differ with regard to their average edge importance ($\overline{IMP_{e}}$). Redundant node features 17 (in case of module 2) and feature 20 (in case of module 3) decrease the average edge importance scores. If we would judge the detected modules based on their module performance $Perf(T_{m})=1$ only, it may result in top-ranked modules including redundant features. On the other hand, if we would exclusively rely on the edge importance $\overline{IMP_{e}}$, we may miss important features. In our example this would make module $\{12, 16, 30\}$ ranked second (see Table 1). In that case, we miss relevant node features associated with node $v_{15}$. The combination of both, $\overline{IMP_{e}}$ and $Perf(T_{m})$ provides a minimal set of most important node features.

**Table 1:** Detected modules from the Barabasi network shown in Fig. 2.

RANK	MODULES	$\overline{IMP_{e}}$	$Perf(T_{m})$	$IMP_{m}$
1	{12, 15, 16, 30}	0.67	1	1.67
2	{12, 15, 16, 17, 30}	0.57	1	1.57
3	{12, 15, 16, 20, 30}	0.56	1	1.56
4	{12, 16, 30}	0.64	0.81	1.45
5	{12, 15, 30}	0.59	0.80	1.39
6	{12, 16, 17, 30}	0.51	0.80	1.31

**Figure 3:**
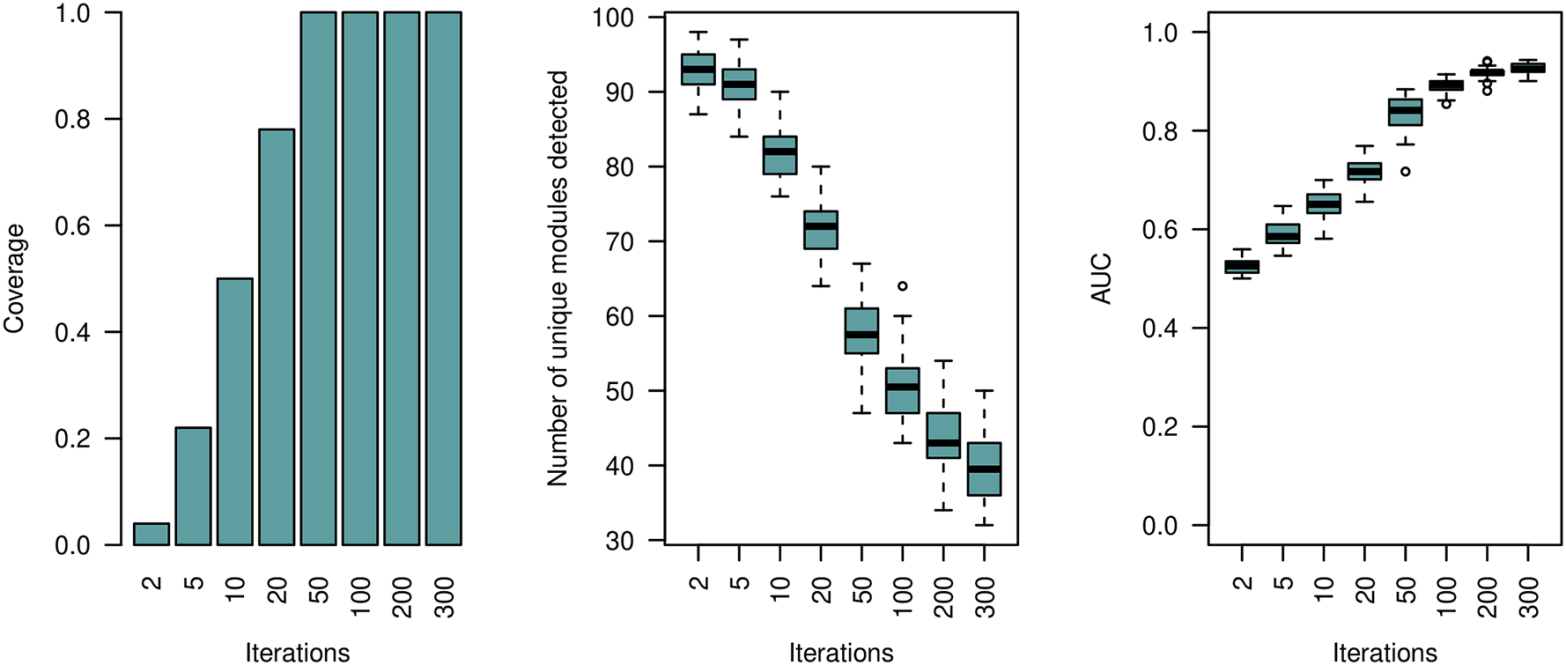
*Single-Modal* simulation results on Barabasi networks. We varied the number of greedy iterations (*n*.*iter*) and calculated the number of times the selected module is ranked first according to our proposed module importance score $IMP_{m}$ (left panel). Displayed are the number of unique modules within the *n*.*tree* module set after termination of the greedy process (middle panel). The *out-of-bag* performance of the Greedy Decision Forest classifier is shown in the right panel. For each run the topology of the Barabasi network, the feature values, and the selected subnetwork is the same. The simulated Barabasi networks comprise of 50 nodes.

In a further analysis we studied the effect of the parameter *niter* on the performance of our module selection method. We have generated a Barabasi network comprising 50 nodes and executed the module selection procedure 100 times. For each run the topology of the Barabasi network is the same, but we varied the selected nodes as well as the binary feature vectors. We report on the top-1 coverage, which in our case is the number of times the module of interest is ranked first. In addition, we report on the number of unique modules out of the *n*.*tree* set after *niter* greedy steps, and the overall *out-of-bag* performance of the Greedy Decision Forest classifier. Results are shown in Fig. 3.

The target network module is consistently ranked first ($coverage=1$) after 50 greedy iterations. Also, the performance of the Decision Forest classifier stabilizes at $AUC~=0.9$ after 50 greedy runs. The number of unique modules decreases with the number of iterations and starts to converge at 200 iterations. We observed that the lower-ranked modules are forming graphs which all include the module ranked first as a sub-graph (similar to what can be seen from Table 1).

Each node may include heterogeneous multi-modal features. This is typically the case in integrative multi-omic studies. Possible features may include gene expression levels, micro-RNA, and DNA Methylation data for the same set of patients. In order to test the general applicability of our approach for these type of applications we define a multi-modal XOR module on a Barabasi network as follows:

13 $$\begin{aligned} Module(V,E):= [f_{a}(\mathbf {v}_{1}) \wedge f_{b}(\mathbf {v}_{2})] \oplus [f_{a}(\mathbf {v}_{3}) \wedge f_{b}(\mathbf {v}_{4})], \end{aligned}$$

where $f(\mathbf {v})$ is the feature vector associated with node $\mathbf {v}$, and *a* refers to the features of the first modality and *b* to the second.

**Figure 4:**
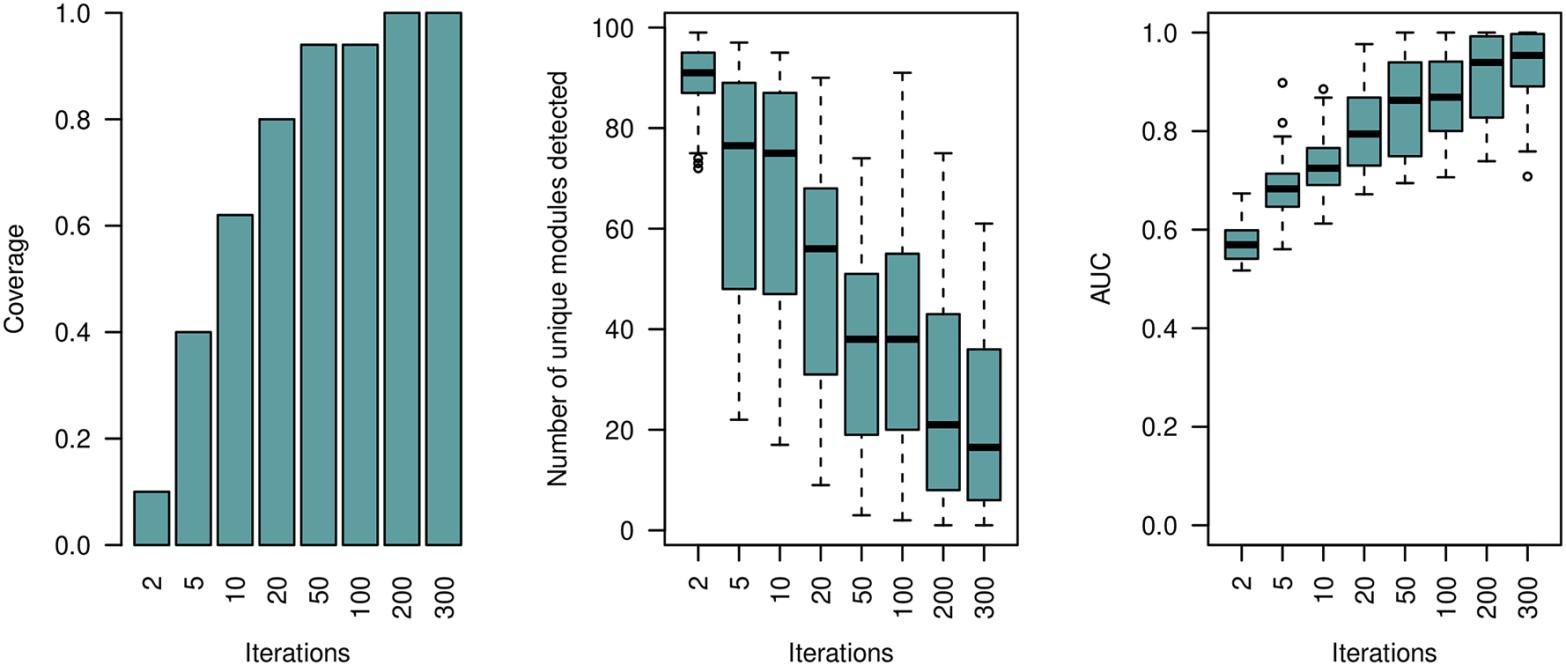
*Multi-Modal* simulation results on *variable* Barabasi networks. We varied the number of greedy iterations (*n*.*iter*) and calculated the number of times the selected module is ranked first according to our proposed module importance score $IMP_{m}$ (left panel). Displayed are the number of unique modules within the *n*.*tree* module set after termination of the greedy process (middle panel). The *out-of-bag* performance of the Greedy Decision Forest classifier is shown in the right panel. For each run the topology of the Barabasi network, the feature values, and the selected subnetwork *varies*. The simulated Barabasi networks comprise of 50 nodes.

Supplementary Fig. 1 shows the results when the Barabasi network structure is the same in all iterations. The results are very similar to what we have seen in the single-modal case (Fig. 3). This observation indicates that the proposed algorithm is fully capable of detecting important sub-networks even when the relevant information is distributed across multiple modalities.

In a additional investigation, we varied the topology of the Barabasi networks, as well as their corresponding binary node features (Fig. 4). Results confirm that the proposed Greedy Decision Forest can be efficiently applied to multi-modal feature inputs. The coverage is comparably high and is 1 starting with 200 greedy iterations (see Fig. 4). However, both, the *out-of-bag* *AUC* values and the number of detected unique modules have a wider distribution (Fig. 4). Thus, ee can conclude that the performance of our proposed algorithms depends on the topology of the Barabasi networks (see Supplementary Fig. 1 versus Fig. 4). It should be noted, that the logical XOR produces balanced outcome classes. We have repeated the same experiment but computing the outcome class using a logical OR and a logical AND. The results are shown in Supplementary Fig. 2 and Supplementary Fig. 3. The Greedy Decision Forest converges to the selected module after 50 greedy iterations in both cases.

### Application to multi-omics TCGA cancer data

We showcase the applicability of our approach on a Protein-Protein Interaction Network (PPI) for the detection of disease modules. The PPI network was retrieved from the STRING database^45^. We only kept high confidence interactions (a *combined score* within the upper 0.95 quantile). We filtered for relevant cancer genes as specified in^46^. This resulted in 13,218 relevant genes and 6,926,452 edges. We enriched each node of the PPI network by multi-omic features from kidney cancer survival and non-survival patients. The omic features were extracted from http://linkedomics.org/^47^. We selected gene expression (mRNA) and DNA Methylation data for the same set of patients.

Two experiments were conducted. First, we investigated the capability of the proposed approach to predict the survival status of patients suffering kidney cancer, to infer potential subnetworks for risk assessments.

In a second experiment, our aim was to detect kidney-specific disease modules. For this analyses we randomly selected patients suffering lung, ovarian, and breast cancer and assigned these patients to a non-kidney cancer group. Here the goal was to detect modules as potential biomarkers specific to kidney cancer. After harmonization with the reduced PPI network, we ended up with 6249 network nodes (genes) for the survival analyses, and 3374 genes for the cancer type experiment (see Table 2).

**Figure 5:**
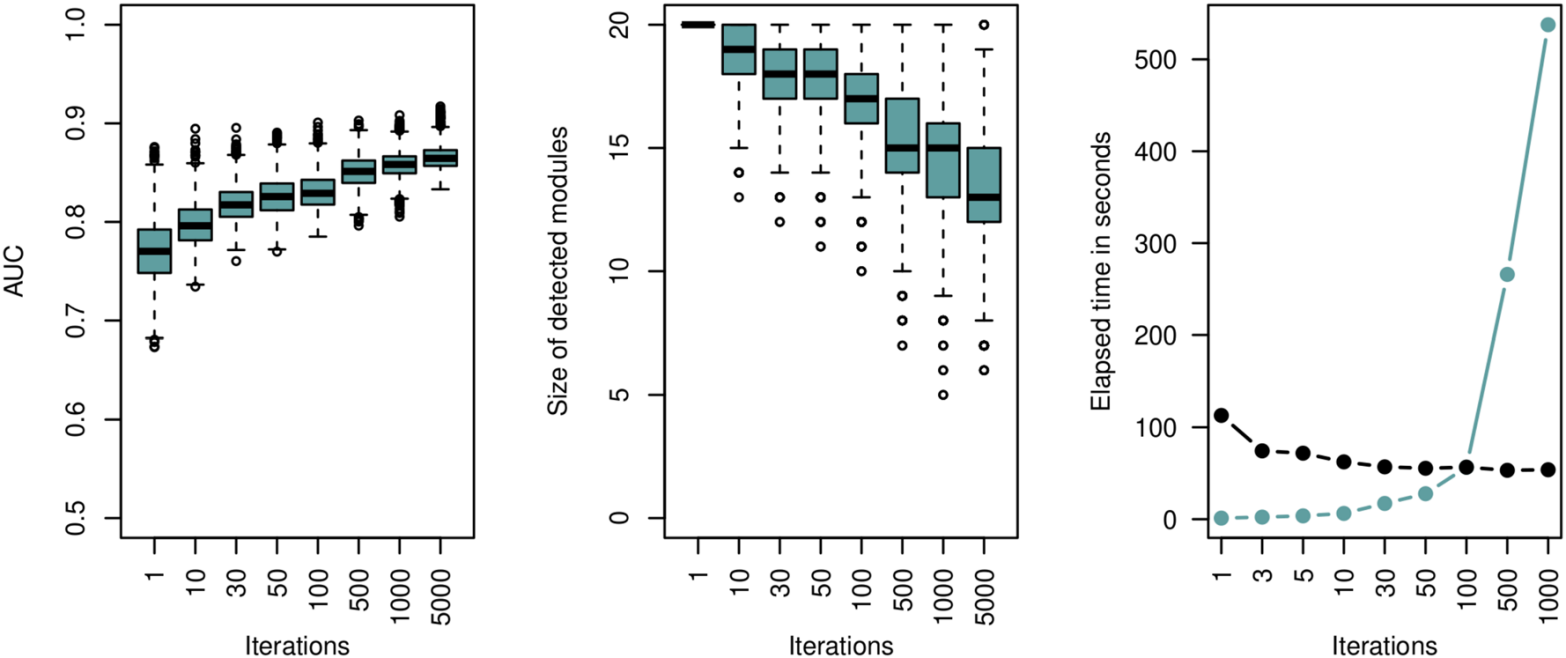
Disease module detection for kidney cancer survival prediction. Shown is the *out-of-bag* accuracy of the detected modules while varying the number of iterations (left panel). The second figure reports on the size of the detected modules. The computational demand of our proposed method is shown in the right panel. The blue line reflects the absolute computational cost in seconds, the black line is the computational cost divided by the number of greedy iterations.

First, we studied whether the GDF does behave similar on real-world cancer data to what we have seen from the synthetic data. Figure 5 shows the size of the inferred modules and the corresponding *out-of-bag* accuracy, while increasing the number of greedy iterations. Also, we show the computational demand of our method (Table 5). The experiment confirms that the decision trees (modules) converge to better performing and smaller modules.

For both experiments we applied a 80–20% train-test split and initiated 500 random walks (n.tree = 500) on the PPI network based on the train set. The number training and test samples are shown in Table 2.

**Table 2:** Experimental data ( survived, *ns* non-survived, *rand* random cancer type).

Experiment	Genes	Patients (train set)	Patients (test set)
Survival status	6249	85 (s), 79 (ns)	18 (s), 24 (ns)
Cancer type	3374	162 (kidney), 158 (rand)	38 (kidney), 42 (rand)

The depth of the random walks, capturing the nodes for tree building, was set to an initial value of $mtry=30$. Thus, we were interested in disease modules comprising less than 30 genes. The derived 500 Decision Trees we then let evolve $n.iter=100$ greedy iterations. The greedy process terminated after 55,000 trees. The predictive performance of our Greedy DF classifier can be obtained from Table 3. It is based on the predictions of the last 500 trees (*n.tree*) of the decision forest, which include the selected modules.

We compared the predictive performance of our Greedy DF classifier with Random Forest (RF), a fully connected Neural Network (NN), and the method implemented within the blockForest^48^ program. For these classifier we do not incorporate any domain-knowledge about the interaction or relatedness of genes. Moreover, we have compared our GDF classifier with a graph neural network approach for disease module detection, which is implemented within the Python package GNN-SubNet^49^ (https://github.com/pievos101/GNN-SubNet). We have repeated the 80–20% train-test split 20 times and report on the min, median, and max accuracy (see Supplementary Table 1; Table 3).

**Table 3:** Performance of the Greedy Decision Forest (min/median/max).

Experiment	Sensitivity	Specificity	Precision	Accuracy
Survival status	0.35/0.60/0.78	0.50/0.65/0.93	0.46/0.61/0.94	0.50/0.64/0.77
Cancer type	0.75/0.87/0.93	0.62/0.73/0.86	0.73/0.79/0.86	0.79/0.85/0.89

We learned that the alternative approaches performed equally good as the proposed Greedy Decision Forest, in terms of model performance. This is an intriguing result since we aim for increased interpretability while maintaining predictive performance.

We conducted an in-depth comparison with a standard random forest approach where no graph structure is incorporated. We varied the number of trees (Supplementary Fig. 5) and the size of the *mtry* set (Supplementary Fig. 4). We could learn that the incorporated domain knowledge via a PPI network does not increase the predictive performance of the classifier. The main focus of this paper, however, is module selection, and classical machine learning approaches do not provide such selective procedure. In contrast to other methods, our DF classifier comprises of decision trees with functional related biological entities and thus may be more interpretable by a domain expert.

For instance, the Greedy Decision Forest selected a module comprising of three genes for the cancer type experiment, namely COG1, COG3, and COG5 (see Supplementary Fig. 6). The module achieved an accuracy of 0.78 on an independent test set (see Table 4). We compared the quality of the selected genes with those obtained from GNN-SubNet, RF, and RF with selected features obtained from the Boruta^21^ program, in terms of classification accuracy and gene ontology enrichment. The results are shown in Table 5. The accuracy of the selected genes is similar for all methods. GO enrichment analysis, however, resulted in highly significant values for GNN-SubNet and our Greedy Decision Forest, indicating a strong signal of common biological functionality of the selected genes. We further investigated the connectivity of the selected modules (see Table 5; Supplementary Fig. 7). Here, we varied the cut-off for the confidence of the PPI edges and report on the pairwise shortest paths from one protein to the other. The PPI shortest path distance between the nodes is clearly higher for the alternative approaches.

**Figure 6:**
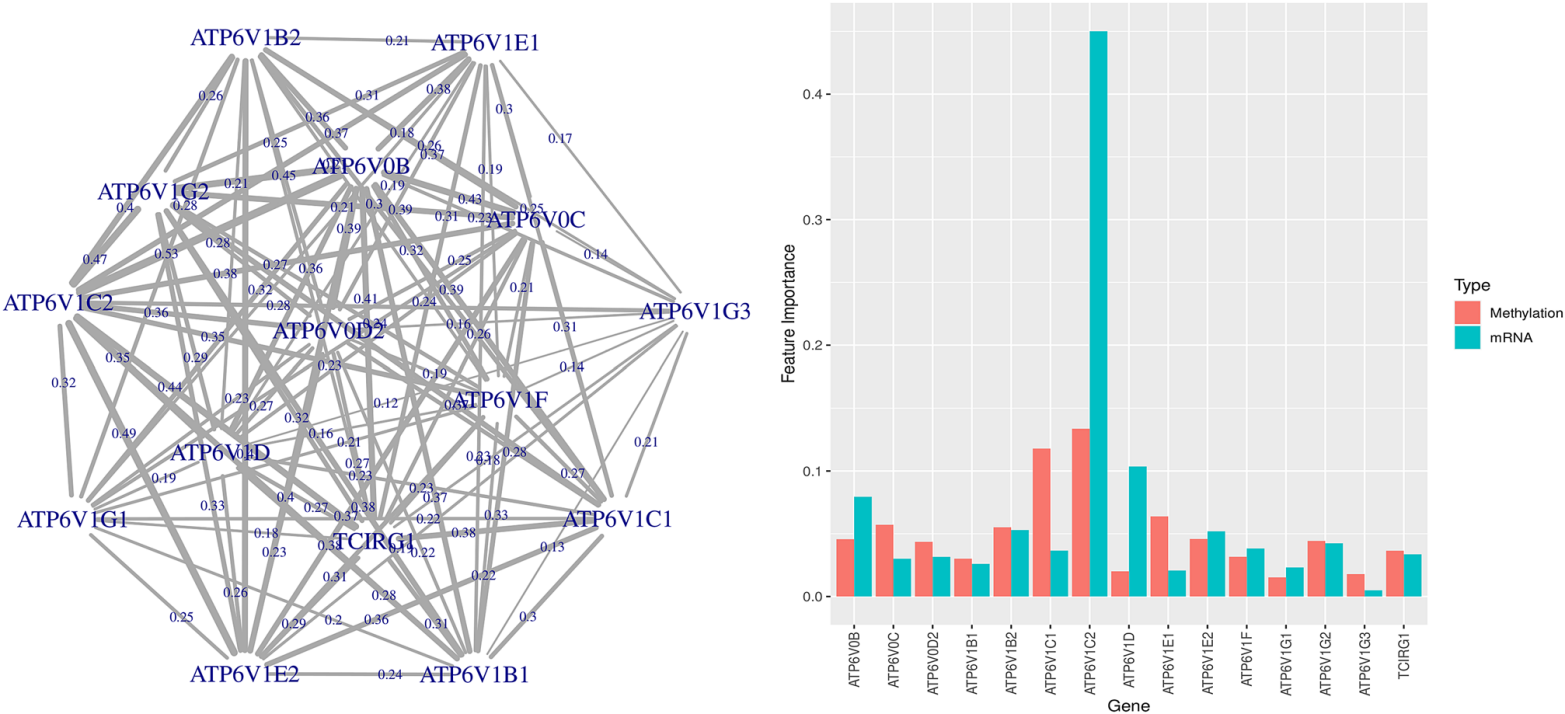
Disease module for classification of kidney cancer survival and non-survival patients. Left panel: disease subnetwork with importance of the edges ($Imp_{e}$), indicated by edge thickness. Right panel: multi-modal importance measures of the involved omics ($IMP_{f}$).

Finally, for the survival analysis, the detected modules/trees include about 10–20 genes. The top-ranked module, according to $IMP_{m}$, comprises 15 genes (see Fig. 6). The module has an *out-of-bag* accuracy of 0.72. The test set performance of that module can be seen in Table 4.

**Table 4:** Performance of the detected module.

Experiment	Size of module	Sensitivity	Specificity	Precision	Accuracy
Cancer type	3	0.85	0.71	0.77	0.78
Survival status	15	0.65	0.74	0.59	0.69

**Table 5:** Cancer type experiment.

Method	Selected genes	Accuracy	PPI distance	GO enrichment
			[0,500,700.900]	*p value*
GDF (ours)	**COG1**, **COG3**, **COG5**	0.78	1,1,1,1	$0.7^{-9}$
GNN-SubNet	**MGAT3**, **MGAT4B**,**MGAT5**, **MGAT5B**	0.79	1,1,2,2	$0.3^{-10}$
RF	CUL2, **LRSAM1**, CCT3	0.81	2,2,2,2	$0.1^{-2}$
RF$_{Boruta}$	**ATP2A1**, CASP2, GMIP, HELQ, IQGAP3	0.81	2,4,4,5	$0.1^{-2}$

Accuracy of the selected kidney cancer (KIRC) genes. GO term enriched genes are highlighted in bold. The PPI Distance reflects the longest shortest path from one node to the other, given a certain threshold for the confidence of the edges.

**Table 6:** Survival experiment.

Method	Selected genes	Accuracy	PPI distance	GO enrichment
			[0,500,700.900]	*p value*
GDF (ours)	**ATP6V1B1, ATP6V1E1, ATP6V1C2, ATP6V1E2, ATP6V0C,**	0.69	1,1,1,2	0
	**ATP6V0B, TCIRG1, ATP6V1B2, ATP6V1D, ATP6V1C1,**			
	**ATP6V1G2, ATP6V1F, ATP6V0D2, ATP6V1G1, ATP6V1G3**			
GNN-SubNet	DMBT1, **NAPSA, SFTPA1**,	0.62	1,3,3,3	$0.4^{-11}$
	**SFTPA2**, SFTPB, SFTPC, **SFTPD**			
RF	SLC22A8, **ACTN2**, CCNF, TMTC2, **KCNQ2**,	0.53	3,4,6,NA	$0.7^{-3}$
	TJP1, GPR84, PRDM8, SERTAD3, NEDD9,			
	TARBP2, **SLC9A3**, CCT2, GTF3C2, RAB11FIP1			
RF$_{Boruta}$	**ACVR2A, AVIL, PYGO2, STK24**, TMTC3	0.64	2,4,6,NA	$0.7^{-3}$

Accuracy of the selected kidney cancer (KIRC) genes. GO term enriched genes are highlighted in bold. The PPI Distance reflects the longest shortest path from one node to the other, given a certain threshold for the confidence of the edges.

The Decision Tree associated with the top-ranked detected module had a high specificity, but the precision of the predictions were moderate. A rather low value for this specific experiment was expected, because the detection of network modules causing a death outcome in patient survival analysis is a hard task.

We compared the results with a standard RF approach, where we selected the top-15 (same number as for the size of the detected module) ranked features according to the in-build random forest impurity scores. We trained a random forest based on these features, and applied the trained model to the test data set. The accuracy of the RF was significantly lower than for our approach, which performed best with an accuracy of 0.69 (see Table 6). The random forest based on the selected features from Boruta had better performance than RF. GO enrichment values were best for GNN-SubNet and our Greedy Decision Forest (GDF) (see Table 6). The connectivity (PPI distances) of the obtained gene sets can be obtained from Table 6 and Supplementary ***Fig. 8. These results suggest, that feature selection based on features organized in modules may perform superior on unseen test data.

The genes forming the detected module point to an interesting biological ATP protein complex. ATP proteins are mitochondrial transport proteins and were recently verified as potential biomarker for kidney survival prognosis^50^. In addition to that, we report on a special importance of the ATP6V1C2 protein for survival prediction (see Fig. 6, right panel). Furthermore, the ATP6V1C2-ATP6V0B protein interaction has the highest edge importance score in that module, with $IMP_{e}=0.52$. This finding may suggest the ATP6V0B gene as an important co-expression partner (Fig. 6, left panel).

A potentially important role of ATP6V1C2 is also reported by the SHAP node importance measured on the test set predictions of the best module (see Supplementary ***Fig. 9, left panel top). DNA Methylation data for that gene does not contribute to the feature importance values (Supplementary ***Fig. 9, left panel bottom), which is consistent with Fig. 6. The previously mentioned ATP6V1C2-ATP6V0B protein interaction is also very important (3rd) based on SHAP (Supp***. Fig. 9, right panel). This edge importance explanation is based on SHAP interaction values^41^ and extends our analysis to feature dependencies derived from domain knowledge.

**Figure 7:**
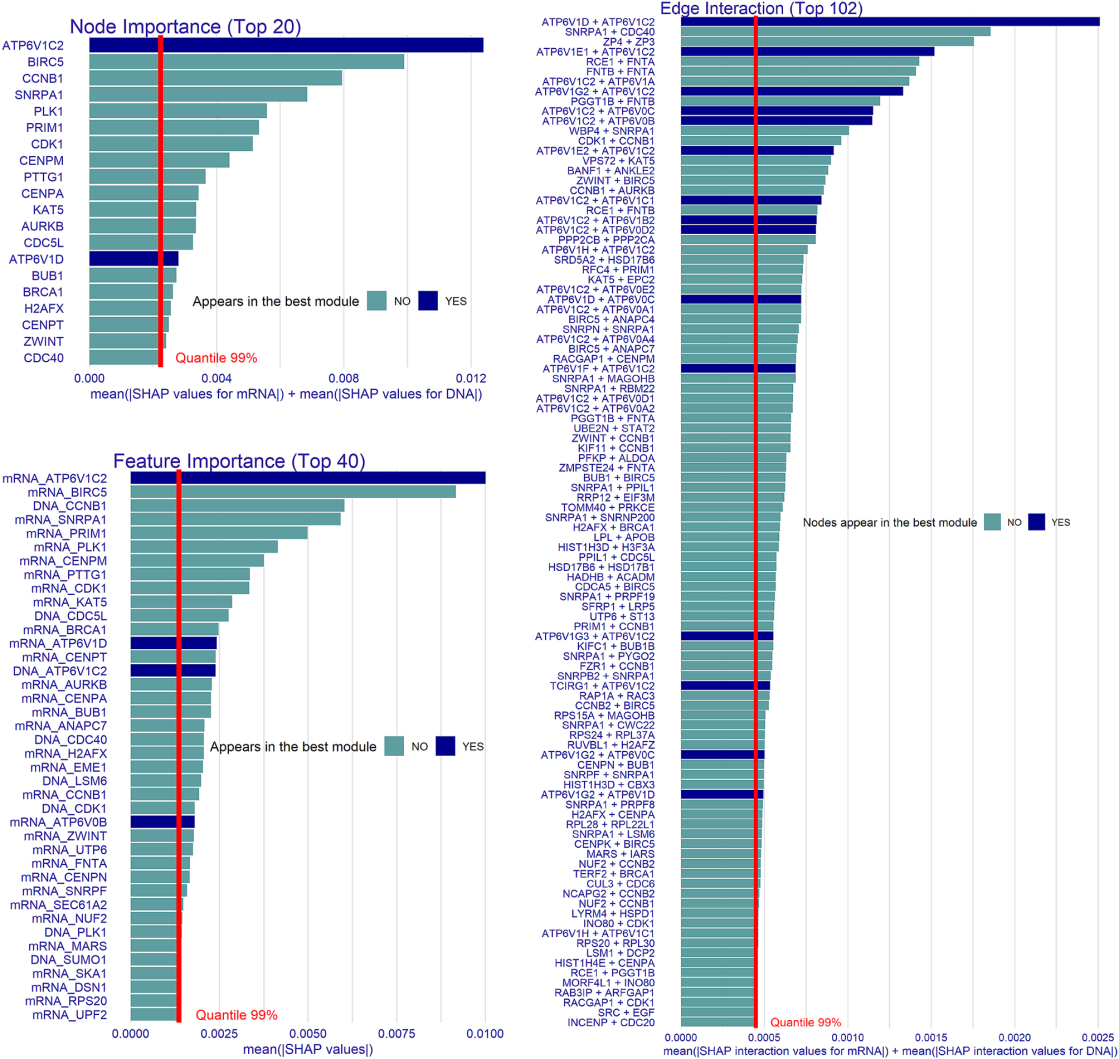
Survival experiment. Explanations of the greedy decision forest classifying kidney cancer survival and non-survival patients. The red lines denote the 99-percentile value of measures on the X-axis, i.e. top 1% of the elements are shown on the Y-axis. Elements appearing in the best decision tree module are highlighted in blue (see Fig. 6). Left: multi-modal SHAP node importance ($SVIMP_f$) and SHAP feature importance ($SVIMP_j$) plots. We attribute differences in the two importance rankings to the multi-omics data structure. **Right**: multi-modal SHAP interaction values aggregated between the feature pairs. Overall, there are about $10^4$ interactions in the model, and we visualize the most important ones.

We contrast SHAP based on the best module (Supp. Fig. 9) with explanations for the GDF classifier consisting of 500 modular decision trees (Fig. 7). Top 1% of the most important elements in GDF are visualized, and the nodes appearing in the best module are highlighted in blue. We see about 10–15% of the best-ranked elements highlighted across the various explanations, which we believe indicates a high performance of the feature selection process. Node importance explanation allows aggregating information over multi-omics data—ATP6V1C2^50^, BIRC5^51^, and CCNB1^52^ appear as most important (Fig. 7, left panel). While the interaction of ATP6V1C2-ATP6V0B is again critical, another interaction between ATP6V1D-ATP6V1C2 becomes the most important feature pair in the GDF (Fig. 7, right panel).

With SHAP values, it is also possible to explain predictions for a single patient in a post-hoc manner. For example, we report on the feature importance of a non-survived patient (see Supplementary Fig. 10). Overall, the distribution of SHAP values for a given observation is highly skewed—there are many (i.e. 99%) features with minimal contributions. Interestingly, the top 5 contributing features are based on gene expression signatures, and ATP6V1C2 is within that set but does not have much influence. High gene expressions within the ATP gene family might contribute to an overall better survival state but might not be used as a predictor for a deadly outcome. We believe that explanatory model analysis incorporating multiple modalities like a graph brings an enhanced view of the current state of knowledge.

## Discussion

In this work, we have proposed a tree-based network module selection algorithm. It can be applied to the scenario where each node is linked to a specific feature vector and any kind of dependency between these features are reflected by the edges of a given domain knowledge network. We have validated our approach on synthetically generated Barabasi Networks. We could show that our approach can be naturally applied to the case of multi-modal input features, where each node comprises feature vectors from multiple modalities.

We showcased the applicability of our approach on a protein–protein interaction (PPI) networks, where each node is enriched by multi-omics features, to detect disease causing modules. We believe we have developed a useful framework which may find wide applications in the future, in all areas of research, but especially within the bio-medical domain. This is because the Greedy Decision Forest for module selection is a highly interpretable approach and thereby decisions it makes, or modules it detects, are traceable with little effort. Deep Learning methods on graphs may solve similar tasks in the future, but are black-box models. Explainable AI methods are further needed to uncover the underlying decision path. In fact, our proposed Greedy Decision Forest may act as a baseline for further developments in that field solving similar problems by the deep learning community.

However, several technical improvements to the proposed algorithm could be made. The PPI network retrieved from the STRING database contains information about the certainty about a functional relationship between two proteins. It is reflected by a “combined score” value, which may change depending on future biological experiments and research findings. Therefore, a weighted random walk could be utilized to explore paths within the PPI network using edge specific certainty scores. Second, the *niter* (number of greedy steps) is a crucial parameter. We not yet provide a stopping criteria. It is similar to the problem of choosing the right number of trees within a Random Forest. We plan to work on a mechanism which monitors convergence during the greedy runs while improving the sampling strategy to accelerate exploration.

## Conclusion

We propose a novel approach for selecting disease modules from multi-omic node feature spaces while including domain-knowledge into the algorithmic pipeline. Our approach can be applied to a variety of possible applications where network structured data form the input and the corresponding nodes are associated with multi-modal numerical feature values. Disease module detection in PPI networks is a typical example of such an application. This work represents a contribution to the field of explainable AI for systems biology. Our proposed Greedy Decision Forest is straightforward to interpret. All decision paths of a detected module are explainable and interpretable by a human domain expert and any contribution of features from different modalities can be retrieved. In comparison with a standard Random Forest we could show that our Greedy Decision Forest increases interpretability while maintaining the predictive performance. Tree-based Shapley values can be utilized for explanations, when our Greedy Decision Forest is utilized as a machine learning classifier. We could showcase, that Shapley values report on biological more relevant patterns when the tree model is learned on PPI networks.

In future work, we will extend our framework for explicit patient survival analyses, taking into account survival status as well as survival times. Furthermore, the analysis of mixed input data will be supported, whereby both categorical and numerical node features of arbitrary dimensions can be considered. While we believe that our method is very useful for systems biology applications, this method is also applicable in other domains, where the underlying data can be structured as a graph and nodes are enriched by feature values.

## Supplementary Information

Supplementary Information.

## Data Availability

The TCGA cancer data underlying this article are available at http://www.linkedomics.org/login.php#dataSource, the PPI networks can be downloaded from the STRING database (https://string-db.org/cgi/download.pl).

## Code availability

We have implemented our GDF approach within the R-package DFNET, freely available on GitHub (https://github.com/pievos101/DFNET). DFNET interfaces with the R-package ranger^53^. Networks are organized and visualized using the R-package igraph^54^. Tree-based Shapley values are calculated using the R-package treeshap^55^.
