# Supplementary material for: Multi-omics disease module detection with an explainable Greedy Decision Forest
Source: Sci Rep. 2022 Oct 7;12:16857. doi: 10.1038/s41598-022-21417-8 (PMC9546860; doi:10.1038/s41598-022-21417-8)
Supplement: Supplementary file 1 — Supplementary Information. [file 41598_2022_21417_MOESM1_ESM.pdf]

Multi-omics disease module detection with an explainable Greedy Decision Forest

Supplementary material

August 4, 2022

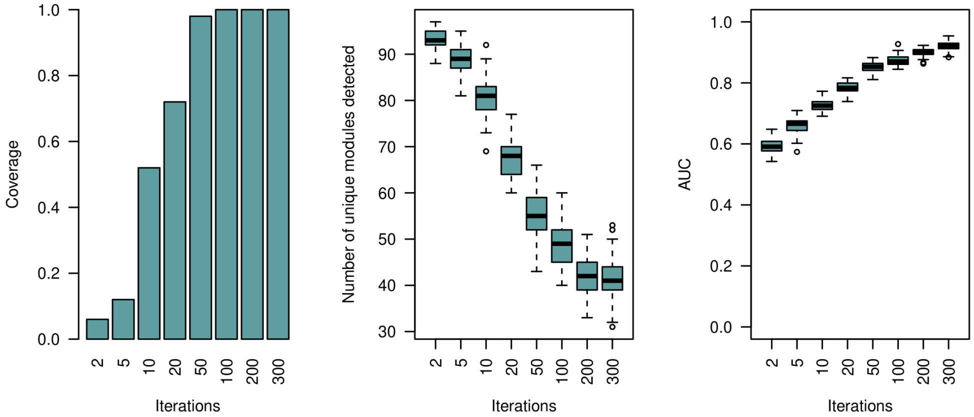

Supplementary Fig. 1: Experiments on synthetic data. *Multi-Modal* simulation results on Barabasi networks. We varied the number of greedy iterations (*n.iter*) and calculated the number of times the selected module is ranked first according to our proposed module importance score IMP_m (left panel). Displayed are the number of unique modules within the *n.tree* module set after termination of the greedy process (middle panel). The *out-of-bag* performance of the Greedy Decision Forest classifier is shown in the right panel. For each run the topology of the Barabasi network, the feature values, and the selected subnetwork is the same. The simulated Barabasi networks comprise of 50 nodes.

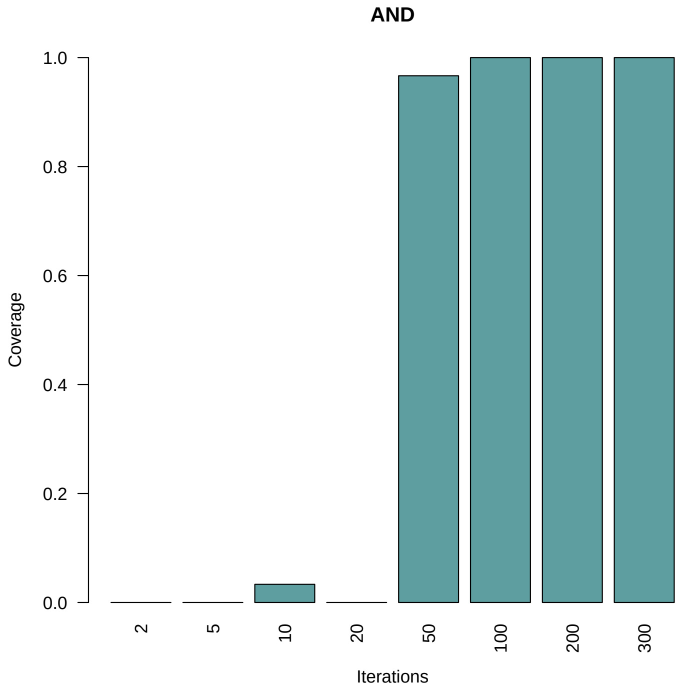

Supplementary Fig. 2: Experiments on synthetic data. Results on synthetic data where the target vector is computed by an logical AND, resulting in an unbalanced class distribution. We subsequentially increased the number of greedy iterations (n.iter) and report on the coverage, which is the number of times the selected module was ranked first.

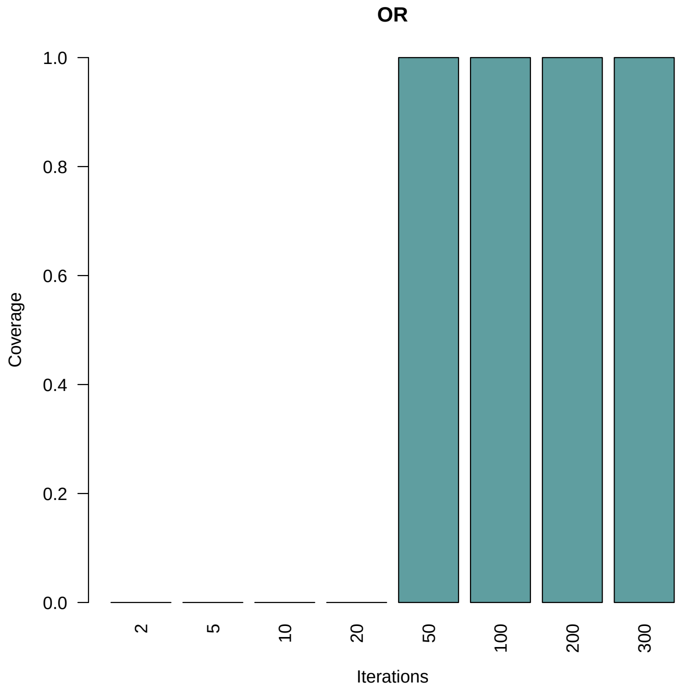

Supplementary Fig. 3: Experiments on synthetic data. Results on synthetic data where the target vector is computed by an logical OR, resulting in an unbalanced class distribution. We subsequentially increased the number of greedy iterations (n.iter) and report on the coverage, which is the number the selected module was ranked first.

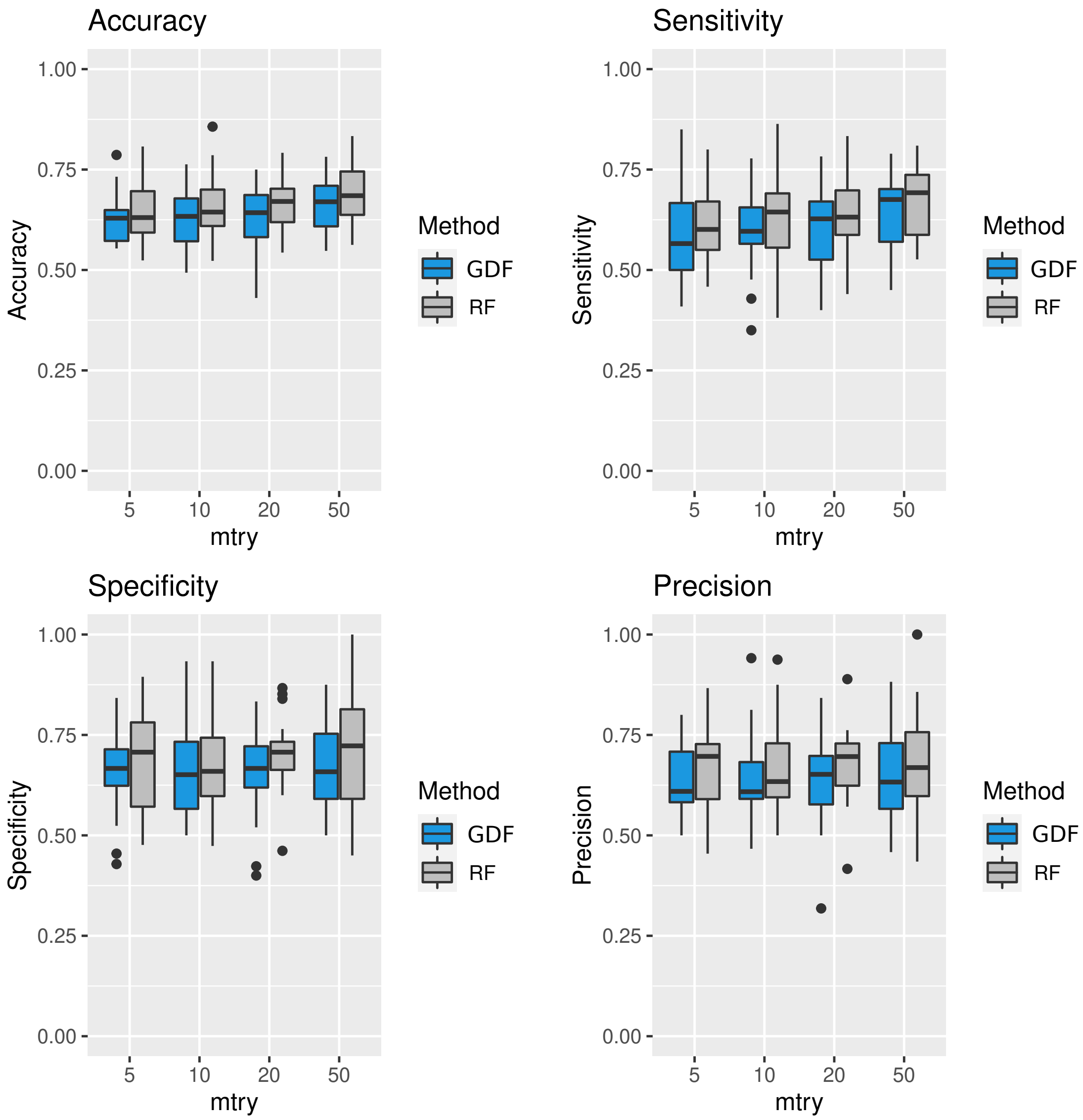

Supplementary Fig. 4: Survival experiment. Comparison of our approach (GDF) with the random forest classifier (RF). We have varied the size of the mtry set and report on the accuracy, sensitivity, specificity, and precision of predicting the survival status of kidney cancer patients. We conducted a t-test to verify whether there is a significant difference between GDF and RF in terms of accuracy. We obtained the following p-values: [0.11, 0.30, 0.40, 0.24, 0.25] for the mtry setting [5, 10, 20, 50]. Thus, according to the t-test there is no significant performance difference between GDF and RF.

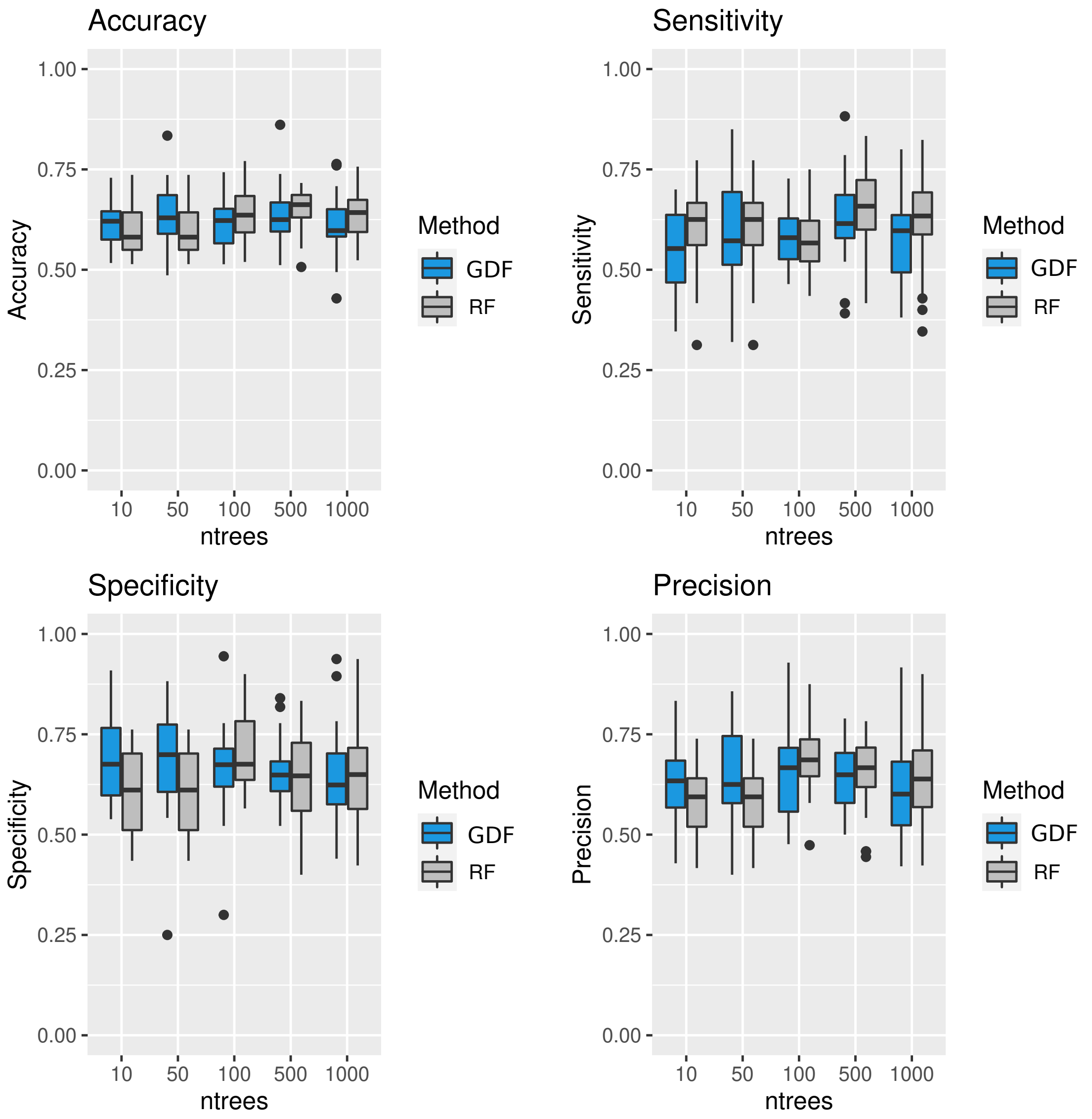

Supplementary Fig. 5: Survival experiment. Comparison of our approach (GDF) with the random forest classifier (RF). We have varied the number of trees (n.trees) and report on the accuracy, sensitivity, specificity, and precision of predicting the survival status of kidney cancer patients. We conducted a t-test to verify whether there is a significant difference between GDF and RF in terms of accuracy. We obtained the following p-values: [0.42, 0.63, 0.44, 0.24, 0.32] for the ntrees setting [10, 50, 100, 500, 1000]. Thus, according to the t-test there is no significant performance difference between GDF and RF.

Supplementary Table 1: Classification accuracy (min/median/max) on the Cancer Type experiment. Comparison with other methods.

**Classifier**	**Cancer**	**mRNA**	**DNA Methylation**	**Multi-omics**
GDF (ours)	KIRC	0.75/0.78/0.84	0.78/0.84/0.86	0.79/0.85/0.89
	BRCA	0.62/0.70/0.78	0.69/0.70/0.83	0.73/0.76/0.83
	LUAD	0.80/0.87/0.96	0.79/0.83/0.88	0.80/0.86/0.91
NN	KIRC	0.82/0.86/0.91	0.82/0.87/0.92	0.82/0.86/0.93
	BRCA	0.65/0.74/0.85	0.71/0.77/0.82	0.56/0.78/0.86
	LUAD	0.81/0.87/0.91	0.83/0.87/0.92	0.82/0.88/0.95
RF	KIRC	0.80/0.83/0.90	0.82/0.87/0.94	0.81/0.88/0.92
	BRCA	0.65/0.76/0.86	0.67/0.79/0.85	0.71/0.78/0.82
	LUAD	0.77/0.87/0.92	0.82/0.89/0.94	0.85/0.88/0.94
GNN-SubNet	KIRC	0.53/0.57/0.76	0.75/0.83/0.91	0.79/0.85/0.91
	BRCA	0.49/0.55/0.65	0.51/0.71/0.81	0.69/0.76/0.85
	LUAD	0.49/0.51/0.59	0.78/0.87/0.94	0.79/0.91/0.95
blockForest	KIRC	0.74/0.79/0.87	0.75/0.81/0.85	0.68/0.81/0.88
	BRCA	0.65/0.70/0.79	0.64/0.73/0.79	0.67/0.73/0.80
	LUAD	0.77/0.86/0.90	0.79/0.86/0.93	0.80/0.86/0.90

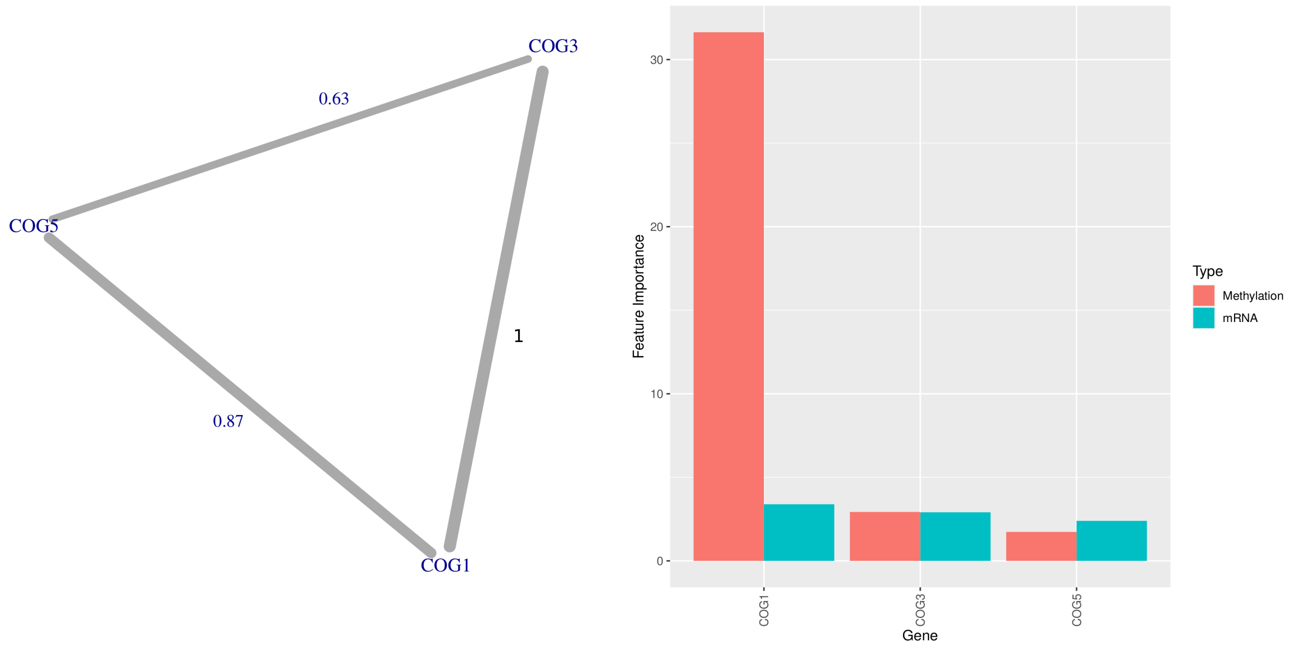

Supplementary Fig. 6: Cancer type experiment. Detected kidney cancer specific disease module (see Table 6 in the main paper). The detected subnetwork is shown, including the edge importances (left panel). The feature importances are shown on the right side.

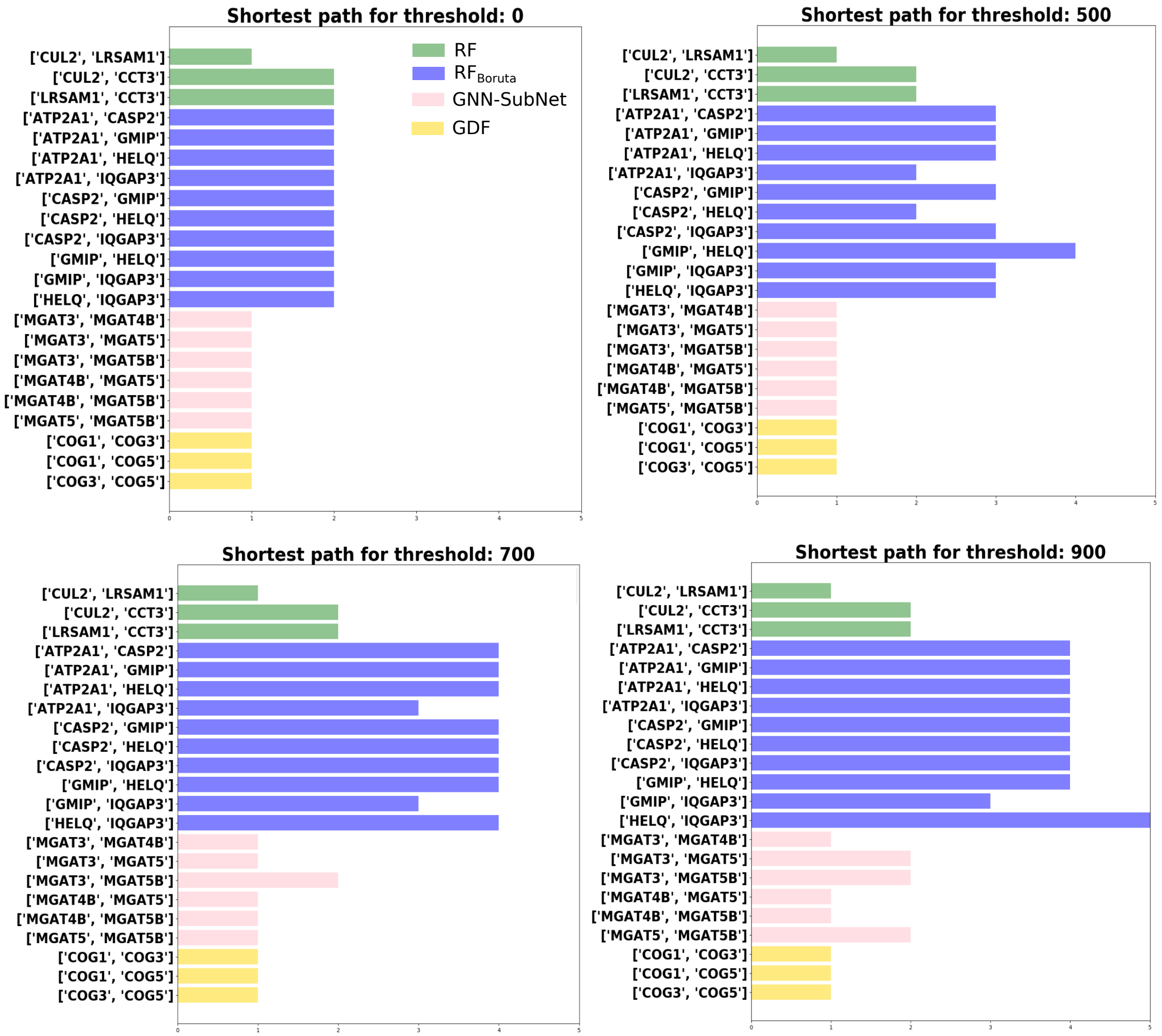

Supplementary Fig. 7: Cancer type experiment. Length of shortest paths between the detected genes. Results are shown for our proposed Greedy Decision Forest (yellow), random forest (green), random forest with feature selection using Boruta (purple), and GNN-SubNet (pink).

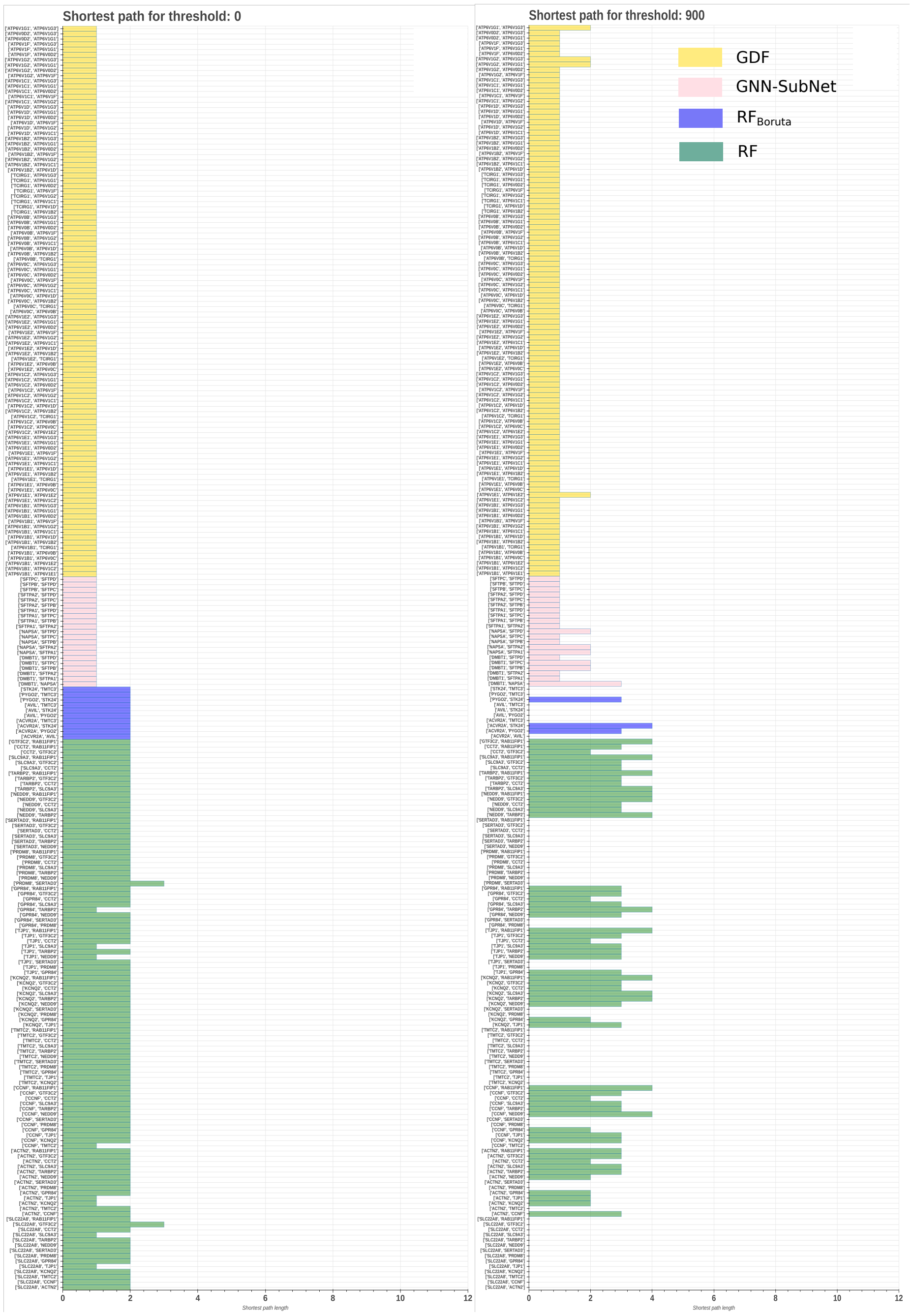

Supplementary Fig. 8: Survival experiment. Length of shortest paths between the detected genes. Results are shown for our Greedy Decision Forest (yellow), random forest (green), random forest with feature selection using Boruta (purple), and GNN-SubNet (pink). Missing bars indicate that no path exist between two nodes within the PPI network for a given threshold.

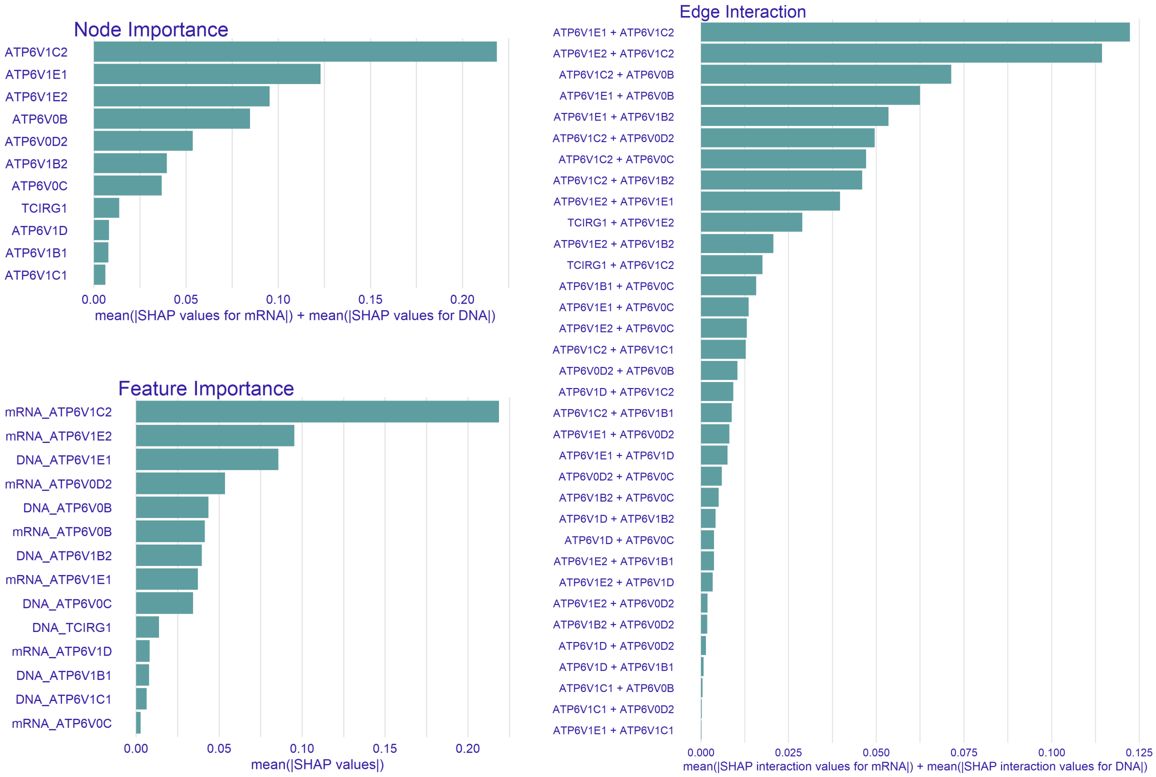

Supplementary Fig. 9: Survival experiment. Explanations of the best decision tree module classifying kidney cancer survival and non-survival patients. **Left**: Multi-modal SHAP node importance ($SVIMP_f$) and SHAP feature importance ($SVIMP_j$) plots. We observe that both measures are consistent, although express importances on a different level of granularity. Node importance aggregation measures become especially useful when increasing the number of modalities in data. **Right**: Multi-modal SHAP interaction values aggregated between the feature pairs. Within the SHAP framework, it is possible to analyze feature interactions in models and compare them with domain knowledge, which gives additional information.

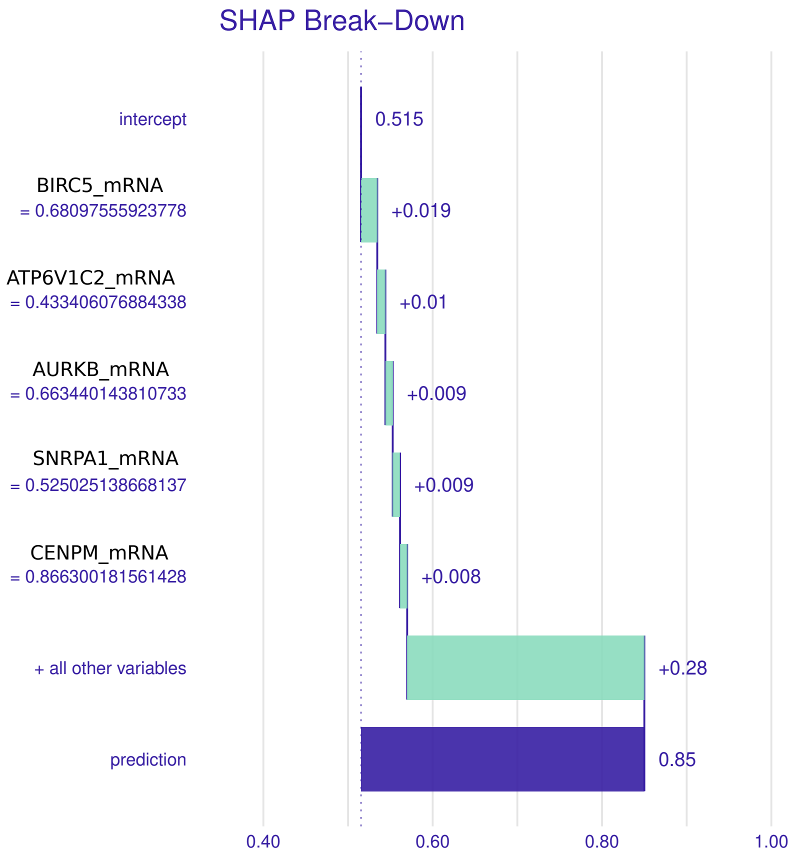

Supplementary Fig. 10: Survival experiment. Exemplary TreeSHAP explanation of a greedy decision forest prediction (0.85) for a single non-survived patient. Intercept denotes the mean model's prediction that equals 0.515. Overall, the distribution of SHAP values for a given observations is highly skewed. There are many (i.e. 99%) features with very little SHAP values while the plot shows top 5 contributing features.
